# Kayexalate-Induced Ischemic Colitis: Revisiting a Forgotten but Preventable Cause of Colonic Necrosis

**DOI:** 10.7759/cureus.118023

**Published:** 2026-10-16

**Authors:** Chorouq Mehdaoui, Chaimae Daoudi, Anass Haloui, Amal Bennani, Hajar Koulali, Ouiam Elmqaddam, Abdelkrim Zazour, Zahi Ismaili, Ghizlane Kharrasse

**Affiliations:** 1 Department of Hepato-Gastroenterology, Mohammed VI University Hospital Center/Mohammed First University, Oujda, MAR; 2 Department of Pathology, Mohammed VI University Hospital Center/Mohammed First University, Oujda, MAR; 3 Department of Pathology, Mohammed VI University Hospital Center/LAPOD (Research Laboratory of Pathology and Digestive Oncology), Faculty of Medicine and Pharmacy, Mohammed First University, Oujda, MAR; 4 Department of Hepato-Gastroenterology, Mohammed VI University Hospital Center/LAPOD (Research Laboratory of Pathology and Digestive Oncology), Faculty of Medicine and Pharmacy, Mohammed First University, Oujda, MAR

**Keywords:** drug-induced gastrointestinal injury, fish scale pattern, ischemic colitis, lower gastrointestinal bleeding, sodium polystyrene sulfonate

## Abstract

Sodium polystyrene sulfonate (SPS), commonly used to treat hyperkalemia, has rarely been associated with severe gastrointestinal injury. We describe a 72-year-old man with chronic kidney disease on long-term SPS therapy who presented with melena and acute kidney injury requiring hemodialysis. Colonoscopy revealed a large ulcerated lesion in the sigmoid colon. Abdominal CT angiography revealed circumferential sigmoid wall thickening with patent mesenteric arteries, raising suspicion of ischemic colitis. Histological examination showed mucosal ulceration and characteristic basophilic, angulated crystals exhibiting a mosaic-like "fish-scale" pattern on hematoxylin and eosin staining, consistent with SPS deposition and supporting the diagnosis of SPS-induced ischemic colitis. SPS was discontinued, and the patient improved with supportive treatment alone. Awareness of this rare complication is important, as early recognition and drug withdrawal may prevent intestinal necrosis, perforation, and death.

## Introduction

Sodium polystyrene sulfonate (SPS), commonly known as Kayexalate, is a potassium-binding resin widely used to manage hyperkalemia, particularly in patients with chronic kidney disease. It acts as a cation-exchange resin, exchanging sodium ions for potassium ions in the gastrointestinal tract, primarily in the colon, thereby promoting fecal potassium elimination [1].

Despite years of use, this drug has been associated with rare but potentially fatal gastrointestinal complications, ranging from ulceration and ischemic colitis to transmural necrosis and perforation [2,3]. Although the exact incidence of SPS-induced ischemic colitis remains unknown, serious gastrointestinal adverse events have been reported in approximately 0.2% of SPS-treated patients within 30 days of exposure [4].

These complications have been reported particularly in vulnerable patients, including those with chronic kidney disease, organ transplant recipients, and patients in the postoperative setting [2]. Other recognized predisposing factors include uremia, concomitant sorbitol administration, and prolonged intestinal transit, which may increase mucosal exposure to the resin [2]. In 2009, the U.S. Food and Drug Administration (FDA) strengthened warnings regarding the risk of intestinal necrosis associated with the concomitant administration of SPS and sorbitol [5].

The cornerstone of diagnosis is the histopathological identification of the characteristic polygonal SPS crystals with a fish-scale pattern [3]. Despite the availability of newer potassium-binding agents, including patiromer and sodium zirconium cyclosilicate, an SPS-associated gastrointestinal injury remains clinically relevant because of its potentially severe consequences and frequently nonspecific presentation [6].

Herein, we report the case of an elderly patient with chronic renal failure who developed ischemic colitis secondary to prolonged Kayexalate therapy. Through this case and a focused literature review, we highlight an under-recognized but preventable complication of SPS therapy and emphasize the importance of early recognition and prompt drug withdrawal.

## Case presentation

A 72-year-old man with a medical history of diabetes mellitus, hypertension, and ischemic heart disease had previously undergone a right nephrectomy reportedly performed for renal abscess complicated by chronic renal failure, with a baseline serum creatinine of 309 µmol/L (3.5 mg/dL) and secondary hyperkalemia treated with oral sodium polystyrene sulfonate, approximately 15g (1 tablespoon) daily, mixed with water, for eight months before his admission for a dialysis emergency for worsening renal function, hyperkalemia, and electrocardiographic abnormalities.

On admission, the patient reported melena and was confused, with a Glasgow Coma Scale (GCS) score of 13/15. He was hemodynamically stable, with a blood pressure of 120/70 mmHg, a heart rate of 97 beats/min, and an oxygen saturation of 97% on room air. Physical examination revealed cutaneous and mucosal jaundice.

Laboratory tests revealed elevated serum creatinine (866µmol/L), hyperkalemia (6.6 mmol/L), and total bilirubin (136.8 µmol/L), with a conjugated fraction of 56.4 µmol/L. Liver function tests showed a cholestatic pattern: gamma-glutamyl transferase (GGT) 835 IU/L, alkaline phosphatase (ALP) 1448 IU/L, alanine transaminase (ALT) 213 IU/L, and aspartate aminotransferase (AST) 350 IU/L. Hemoglobin decreased from 117 to 79 g/L (Table 1). Viral hepatitis serologies for hepatitis B virus (HBV) and hepatitis C virus (HCV) were negative.

**Table 1:** Comparison of laboratory tests before (Day 0) and after hemodialysis (Day 1) and red blood cell transfusion GGT: gamma-glutamyl transferase; ALP: alkaline phosphatase; ALT: alanine transaminase; AST: aspartate aminotransferase

Laboratory tests	Initial values (Day 0)	After hemodialysis and transfusion (Day1)	Reference ranges
Serum creatinine (µmol/L)	866	522	64-111
Potassium (mmol/L)	6.6	3.7	3.5-5.1
Hemoglobin (g/L)	79	90	130-180
Total bilirubin (µmol/L)	136.8	34.2	3.4-20.5
GGT (U/L)	835	298	12-64
ALP (U/L)	1448	1128	40-150
ALT (U/L)	213	103	0-55
AST (U/L)	350	88	5-34

Following hemodialysis and transfusion of four units of red blood cells, the patient stabilized, with a GCS score of 15/15. Laboratory values showed serum creatinine 522 µmol/L, potassium 3.7 mmol/L, hemoglobin 90 g/L, and bilirubin 34.2 µmol/L, suggestive of hepatic dysfunction. Abdominopelvic CT angiography demonstrated no significant atheromatous arterial lesions and patent mesenteric arteries, but revealed circumferential sigmoid colonic wall thickening measuring 7-8 mm, suggestive of nonspecific colitis (Figure 1). These findings made an occlusive mesenteric arterial etiology unlikely, although non-occlusive ischemic injury could not be excluded, secondary to renal impairment.

**Figure 1:**
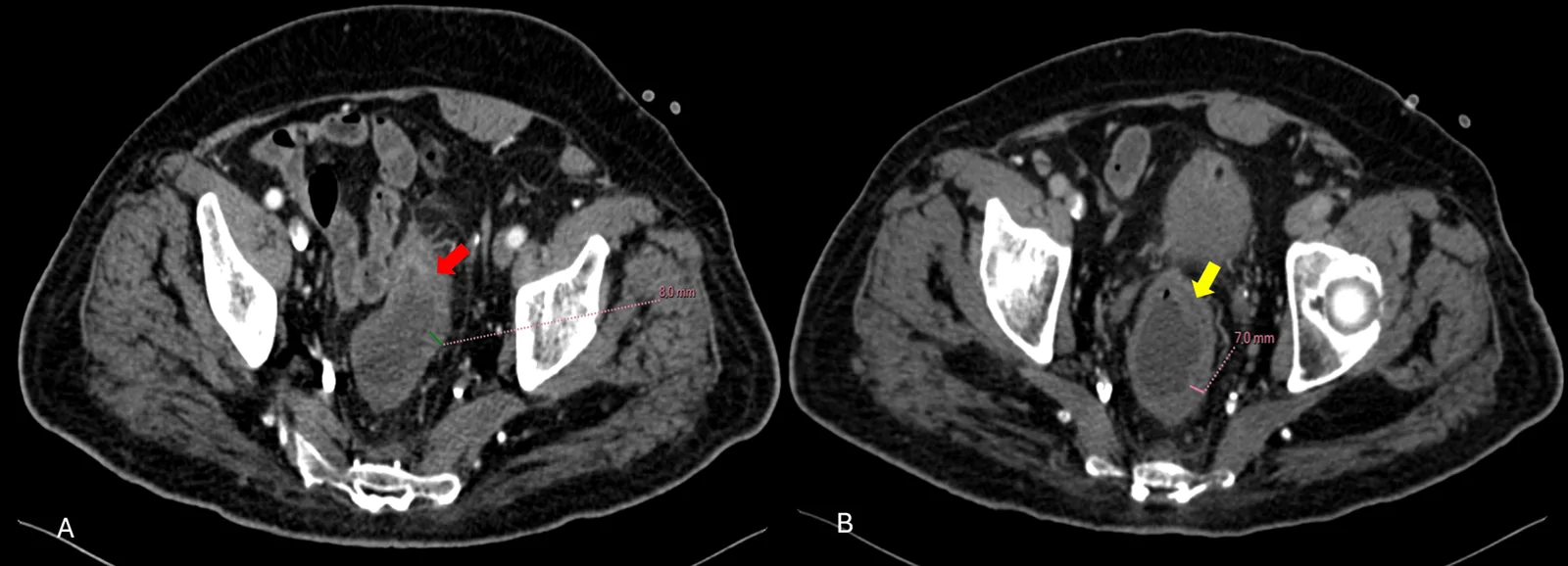
Abdominopelvic CT angiography findings Axial contrast-enhanced CT images obtained during the arterial phase (A) and the portal venous phase (B). The portal venous phase demonstrates circumferential sigmoid colonic wall thickening (yellow arrow), while the arterial phase shows preserved mesenteric arterial patency without evidence of arterial occlusion (red arrow).

Upper gastrointestinal endoscopy revealed no evidence of recent bleeding but showed erythematous, blanching gastritis. Colonoscopy revealed no ileal lesions. In the cecum, multiple diverticula contained stigmata of recent bleeding, suggesting a probable diverticular origin of melena. A large ulcerative lesion, measuring approximately 5 cm in length, with congested edges and areas of granulation tissue, was identified in the sigmoid colon (Figure 2). Given its distal location, which is rarely associated with melena, the sigmoid ulceration was considered a likely incidental endoscopic finding rather than the primary source of gastrointestinal bleeding. Nevertheless, its potential contribution to bleeding could not be entirely excluded.

**Figure 2:**
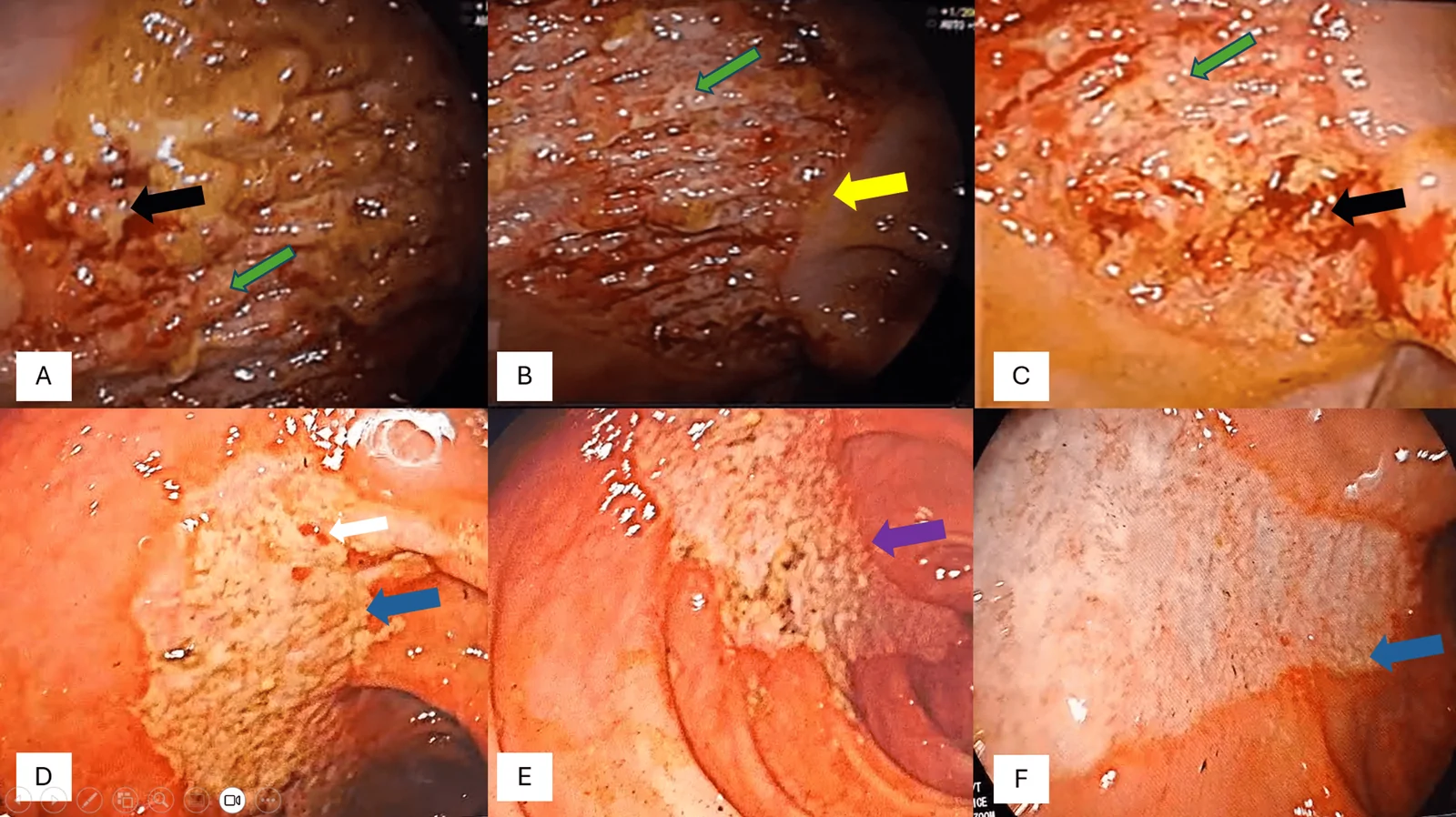
Colonoscopic findings at baseline (D1) and 15 days after kayexalate discontinuation (D15) (A-C) Day 1 (D1): Colonoscopic images showing extensive, irregular, and confluent ulcerations with an endoscopic appearance suggestive of ischemic colitis (yellow arrows), covered with fibrinous and necrotic exudates (green arrows), associated with active bleeding and surrounded by erythematous, friable mucosa (black arrows). Lesions involve the proximal sigmoid colon (A), mid-sigmoid colon (B), and distal sigmoid colon near the sigmoid-descending colon junction (C), consistent with severe ischemic colitis associated with sodium polystyrene sulfonate (Kayexalate) use. (D-F) Day 15 (D15): Follow-up rectosigmoidoscopy images obtained 15 days after Kayexalate discontinuation, demonstrating marked mucosal healing (purple arrow), with residual fibrin-covered ulcerations (blue arrows) and areas of epithelial regeneration (white arrows). These findings involve the proximal sigmoid colon (D), mid-sigmoid colon ( E), and distal sigmoid colon near the sigmoid-descending colon junction (F), consistent with progressive mucosal repair following Kayexalate withdrawal.

Ischemic colitis was initially considered, along with differential diagnoses including neoplastic lesions, medication-induced colitis, infectious colitis (particularly cytomegalovirus (CMV) and *Clostridioides difficile*), and a solitary colonic ulcer.

Abdominopelvic CT angiography demonstrated no significant atheromatous arterial lesions and patent mesenteric arteries, but revealed circumferential sigmoid colonic wall thickening measuring 7-8 mm, suggestive of nonspecific colitis (Figure 1). These findings made an occlusive mesenteric arterial etiology unlikely, although non-occlusive ischemic injury could not be excluded.

Viral serological testing performed during the acute episode revealed positive CMV IgG (>250 AU/mL) with negative CMV IgM (index: 0.13), as well as positive Epstein-Barr virus (EBV) VCA IgG (index: 26.71) with negative EBV VCA IgM (index: 0.05). These findings were consistent with previous viral exposure without serological evidence os acute primary infection.

Given the possibility of drug-induced colonic injury, a detailed medication review was undertaken. The patient’s regular medications included sodium polystyrene sulfonate (approximately 15 g/day), acetylsalicylic acid (75 mg/day), furosemide (40 mg/day), calcium carbonate (500 mg/day), cholecalciferol (100,000 IU every two weeks), iron (14 mg/day) combined with ascorbic acid (180 mg/day), omeprazole (20 mg/day), spironolactone (100 mg/day), and amlodipine (10 mg/day) and magnesium hydroxide (3 g/day) for chronic constipation, which worsened following sodium polystyrene sulfonate initiation. No exposure to sevelamer or cholestyramine was identified in the history of medication.

Sigmoid biopsies revealed extensively ulcerated colonic mucosa with a fibrinoleukocytic coating. The glands showed architectural abnormalities, including cystic dilatation, distortion, and focal scalloping, with a hyperplastic epithelium. Epithelial cells showed regenerative changes, with ovoid nuclei, vesicular chromatin, fine nucleoli, and preserved mucosecretion, along with a few scattered mitotic figures.

The lamina propria contained a dense polymorphic infiltrate composed of lymphocytes, plasma cells, histiocytes, and neutrophils. Foci of cryptitis were observed without crypt abscesses. There was no evidence of dysplasia, malignancy, or solitary ulcer. No characteristic CMV-associated “owl’s-eye” intranuclear inclusions were identified on routine histological examination.

Importantly, polygonal basophilic crystals characteristic of Kayexalate were identified within the ulcerative foci (Figure 3).

**Figure 3:**
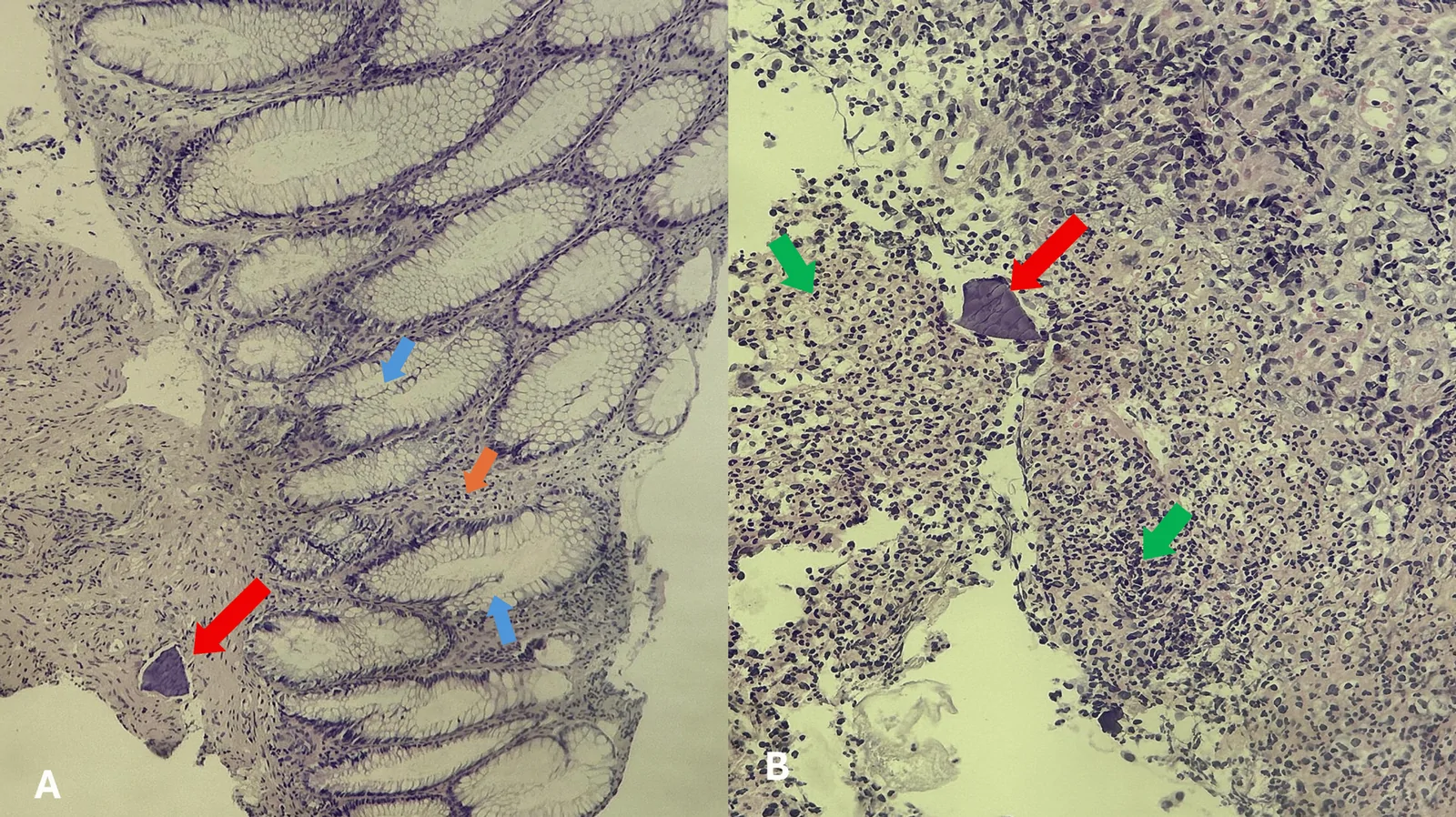
Histopathological findings of sodium polystyrene sulfonate-associated colonic injury (A) Medium-power view of the colonic mucosa showing signs of chronic colitis with crypt architectural distortion (blue arrow), mononuclear inflammatory infiltrate in the lamina propria (orange arrow), along with basophilic crystals of Kayexalate (red arrows) (Hematoxylin-Eosin-saffron coloration, magnification x10). (B) High-power view of the colonic mucosa showing the basophilic, polygonal “fish-scale” crystals of sodium polystyrene sulfonate (Kayexalate) embedded within an area of mucosal ulceration, surrounded by dense inflammatory cells and necrotic debris (green arrow) (Hematoxylin-Eosin-saffron coloration, magnification ×20).

These angulated crystals exhibited a characteristic mosaic-like “fish-scale” pattern on hematoxylin and eosin staining, strongly suggestive of SPS deposition. Polarized light examination was not performed; therefore, their birefringence properties could not be directly assessed. Nevertheless, the characteristic crystal morphology, prolonged SPS exposure, and absence of sevelamer or cholestyramine use supported the diagnosis of SPS-associated colonic injury.

The Naranjo adverse drug reaction probability scale was applied to assess the relationship between SPS exposure and the sigmoid ulceration, yielding an estimated score of 4, consistent with a possible adverse drug reaction. Taken together, the characteristic histopathological findings and prolonged exposure to SPS favored SPS-associated colonic injury rather than establishing definitive causation, prompting withdrawal of Kayexalate.

Fifteen days after Kayexalate discontinuation (D15), the patient showed significant clinical improvement, with complete resolution of melena and marked reduction in asthenia. No episodes of rectal bleeding were reported throughout the clinical course. Hemoglobin levels increased from 7.9g/dL at baseline to 10g/dL on D15. Follow-up rectosigmoidoscopy revealed marked mucosal healing, with a reduction in the extent and severity of the ulcerative lesions. Residual superficial ulcerations covered with whitish fibrinous exudates were observed, along with areas of epithelial regeneration, consistent with progressive mucosal repair following Kayexalate withdrawal (Figure 1).

Although the precise source of the initial gastrointestinal bleeding could not be definitively established, the cecal diverticula were considered the most plausible origin of melena. In contrast, the sigmoid ulceration represented a distinct, likely incidental finding strongly associated with prolonged SPS exposure, as supported by its characteristic histopathological features and subsequent mucosal healing following drug discontinuation.

## Discussion

SPS is a cation-exchange resin approved by the Food and Drug Administration in 1958 that has been used for decades in the management of hyperkalemia, particularly in patients with chronic kidney disease [2]. When administered orally, it releases sodium ions in the acidic environment of the stomach, binds hydrogen ions, and subsequently exchanges hydrogen ions for potassium ions in the small and large intestines [2,7].

SPS was historically combined with sorbitol to prevent constipation and fecal impaction [8]. However, growing evidence suggests that SPS itself can cause intestinal necrosis, independent of sorbitol. Early experiments by Lillemoe et al. in rats showed that colonic necrosis occurred only in animals receiving SPS combined with sorbitol or sorbitol alone, whereas rats treated with SPS alone did not develop this complication [5]. In contrast, Ayoub et al. subsequently demonstrated that 33% sorbitol alone did not induce colonic necrosis in rats, whereas SPS exposure was associated with intestinal injury, suggesting that the resin itself may contribute to mucosal damage [8]. Although the exact mechanism of SPS-induced intestinal injury remains unclear, direct mucosal toxicity and local mechanical irritation related to crystal deposition have been proposed [9]. These effects may be further aggravated by impaired intestinal perfusion, particularly in patients with chronic kidney disease [2,9].

Although the true incidence of SPS-related gastrointestinal injury remains difficult to establish, severe gastrointestinal adverse events have been consistently reported in the literature [2,7]. In their systematic review, Harel et al. identified 58 cases of gastrointestinal injury associated with SPS, occurring both with and without sorbitol. The colon was the most frequently involved site, transmural necrosis was the predominant histopathological lesion, and mortality reached 33% among the reported cases [2,10]. McGowan et al. further reported that SPS-induced intestinal necrosis may occur even in patients without end-stage renal disease or a history of major surgery [2]. More recently, Di Rienzo et al. published a meta-analysis of gastrointestinal histopathological lesions associated with polystyrene sulfonate and sevelamer. Their findings highlighted that gastrointestinal complications related to these resins are uncommon but can have severe, potentially fatal consequences. Abdominal pain (55.3%) and gastrointestinal bleeding (43.5%) were the most frequently reported clinical manifestations [11]. The colon was the most frequently affected site, accounting for 75.9% of cases, followed by the small intestine (14.8%), stomach (8.9%), esophagus (7.6%), rectum (7.6%), and, less frequently, the duodenum (4.2%). Histopathological findings included ulceration (58.2%), necrosis (42.6%), perforation (24.5%), and isolated inflammation (14.4%). The overall mortality rate was 19.8% among the cases included in the analysis [11]. Furthermore, polystyrene sulfonate was significantly associated with a higher frequency of severe outcomes, including necrosis, perforation, and death, compared with sevelamer [11].

Because these resins are not absorbed during gastrointestinal transit, their prolonged contact with the colonic mucosa may partly explain the predominance of colonic injury compared with other segments of the gastrointestinal tract [11]. The rectum, however, is often relatively spared, probably owing to its dual blood supply, which provides greater protection compared with the relatively poorer vascularization of the colon [12]. Other factors may also contribute, including gastrointestinal dysmotility associated with chronic kidney disease and the presence of diverticular pouches, which are less common in the small intestine [8]. Patients who have recently undergone organ transplantation may be particularly vulnerable, as immunosuppressive drugs can impair mucosal defense and healing [12]. Similarly, patients with diabetes or hypertension have been reported to be at increased risk of necrosis or perforation compared with those with isolated nephropathy [11].

Altogether, this suggests that SPS crystals alone are not sufficient to induce mucosal injury. Rather, gastrointestinal damage may occur when predisposing conditions disrupt the intestinal barrier, allowing the insoluble resin matrix to erode the mucosa [13]. In our case, recent nephrectomy, hypertension, diabetes mellitus, and the presence of multiple right colonic diverticular pouches may have been contributing risk factors for the development of Kayexalate-induced ischemic colitis.

Clinically, SPS-associated colitis presents nonspecific manifestations and may mimic other causes of acute colitis [9]. The two most prominent presentations are abdominal pain observed in more than half of cases (≈55%) and gastrointestinal bleeding (≈43%), both of which may rapidly progress to life-threatening complications, including extensive intestinal necrosis and perforation [7]. Other reported symptoms include diarrhea, nausea, vomiting, and abdominal distension [9]. Although some patients present with relatively mild symptoms, severe cases may progress to extensive intestinal necrosis, perforation, or death [11].

Endoscopic findings are highly variable and nonspecific [9,14]. They may range from mucosal erythema, edema, erosions, and superficial ulcerations to more severe presentations, including large or bleeding ulcerations, pseudomembranes, ischemic-appearing mucosa, strictures, pseudotumoral masses, and even frank necrosis involving different segments of the gastrointestinal tract [8,12]. Ulcerations may be extensive and associated with mucosal necrosis or hemorrhage [9]. However, endoscopic findings alone are insufficient to establish causality, as SPS-related injury may closely resemble ischemic colitis, infectious colitis, inflammatory bowel disease, or other forms of drug-induced colitis [8]. Therefore, medication history and histopathological examination, particularly the identification of characteristic resin crystals, are essential for supporting the diagnosis [9,14].

Histopathological findings vary from mild inflammation to extensive necrosis. Microscopically, mucosal ulceration, edema of the lamina propria and submucosa, associated with a dense polymorphous inflammatory infiltrate, are frequently observed, reflecting segmental ischemia and necrosis [9,14]. The diagnostic hallmark is the identification of characteristic SPS crystals within gastrointestinal tissue specimens [9]. A comprehensive review of 135 cases of gastrointestinal injury reported frequent histological identification of resin crystals [9]. These crystals may be found adherent to ulcer bases, embedded in pseudomembranes, or even on the serosal surface after perforation [14]. On hematoxylin-eosin staining, they appear as basophilic, polygonal crystals [9,14]. They are red with periodic acid-Schiff (PAS) and black-red with acid-fast stains, refractile but non-polarizable, described as fishscale-like SPS crystals [15].

Management is based on the immediate discontinuation of SPS and supportive care [2,9]. Although observational studies suggest that SPS may lower serum potassium levels, its use has been associated with a risk of severe bowel injury and death [2]. Given the availability of alternative potassium-binding agents, including patiromer and sodium zirconium cyclosilicate, the potential benefits of SPS should be weighed against its gastrointestinal risks [16]. In severe or life-threatening hyperkalemia, intravenous insulin with glucose and hemodialysis remain important therapeutic options, depending on the clinical context; bicarbonate may be considered in selected patients with metabolic acidosis [16].

The main characteristics of previously published case reports and case series of SPS-induced gastrointestinal ischemic injury are summarized in Table 2.

**Table 2:** Published reports of sodium polystyrene sulfonate (SPS)-induced gastrointestinal ischemic injury

References	Age/Context	Route/Co-administration	Delay	Segment	Histology/main lesion	Management and outcome
Wootton 1989 (Ann Intern Med) [17]	Renal transplant recipients	SPS-Sorbitol enemas	<24h	Colon	Colonic necrosis	Recommended avoidance of SPS-sorbitol enemas in transplant recipients
Scott 1993 (Dis Colon Rectum) [12]	Renal transplant recipient	SPS-sorbitol enemas	Rapid onset	Colon (near-total involvement)	Colonic Necrosis	Severe outcome, historical warning regarding SPS-sorbitol enemas
McGowan 2009 (South Med J) [7]	Mixed (case series)	SPS frequently in sorbitol	3hours -11 days	Intestine, predominantly colon	Intestinal necrosis	Severe outcomes, including 4 deaths
Krishnamurthy 2020 (BMJ) [18]	57-year-old patient with diabetic ketoacidosis	Prior SPS enema	Delayed presentation	Recosigmoid	SPS crystals, colitis, rectal stricture	Severe stenosing complication, surgical management planned
Singhania 2020 (Clinical case reports) [19]	Patient receiving multiple medications	SPS plus activated charcoal	Rapid onset	Small intestine	Intestinal necrosis	Warned against co-administration of SPS and activated charcoal.
Attieh 2025 (ID cases) [20]	Patient without chronic kidney disease	Single dose of Kayexalate	24-48 hours	Colon	Exacerbation of infectious colitis with SPS crystals	Improvement after withdrawal and supportive/infection management.

In our case, the diagnosis of Kayexalate-induced ischemic colitis was supported by the clinical context, exposure to SPS, compatible endoscopic lesions, and histological identification of characteristic resin crystals within ischemic colonic injury. The drug was therefore immediately discontinued. Following SPS withdrawal, the patient showed marked clinical improvement, accompanied by substantial mucosal healing that was confirmed by follow-up rectosigmoidoscopy performed 15 days after treatment discontinuation.

## Conclusions

In conclusion, although Kayexalate has long been considered a standard therapy for hyperkalemia, it may cause severe gastrointestinal injury, including ischemic colitis and colonic necrosis. Our case illustrates the importance of recognizing Kayexalate-associated ischemic colitis, supported by characteristic histopathological findings and favorable endoscopic evolution following drug withdrawal. As the diagnosis of this entity relies on clinical, endoscopic, and histopathological findings, early recognition is crucial. Clinicians should maintain a high index of suspicion in at-risk patients and discontinue the drug at the first signs of gastrointestinal symptoms. In the era of safer therapeutic alternatives, the indication for SPS should be carefully weighed, particularly in patients with gastrointestinal vulnerability or prolonged exposure.
